# Prevention of diabetes in overweight/obese children through a family based intervention program including supervised exercise (PREDIKID project): study protocol for a randomized controlled trial

**DOI:** 10.1186/s13063-017-2117-y

**Published:** 2017-08-10

**Authors:** Lide Arenaza, María Medrano, María Amasene, Beatriz Rodríguez-Vigil, Ignacio Díez, Manuel Graña, Ignacio Tobalina, Edurne Maiz, Edurne Arteche, Eider Larrarte, Inge Huybrechts, Catherine L. Davis, Jonatan R. Ruiz, Francisco B. Ortega, Javier Margareto, Idoia Labayen

**Affiliations:** 1 Nutrition, Exercise and Health Research group, Elikadura, Ariketa Fisikoa eta Osasuna, ELIKOS group, Department of Nutrition and Food Science, University of the Basque Country, UPV/EHU, Vitoria-Gasteiz, Spain; 2 Department of Magnetic Resonance Imaging, Osatek, University Hospital of Alava (HUA), Vitoria-Gasteiz, Spain; 3 Paediatric Endocrinology Unit, University Hospital of Araba (HUA), Vitoria-Gasteiz, Spain; 4 Computational Intelligence Group, University of the Basque Country, UPV/EHU, Donostia, Spain; 5 Department of Nuclear Medicine, University Hospital of Araba (HUA), Vitoria-Gasteiz, Spain; 6 Department of Personality, Assessment and Psychological Treatment, University of the Basque Country, UPV/EHU, San Sebastián-Donostia, Spain; 7 Department of Radiology, University Hospital of Araba (HUA), Vitoria-Gasteiz, Spain; 8 Technological Services Division, Health and quality of life, TECNALIA, Miñano, Spain; 9 Nutrition and Metabolism Section, International Agency for Research on Cancer, World Health Organization, Lyon, France; 10 Georgia Prevention Institute, Medical College of Georgia, Augusta University, Augusta, GA USA; 11 PROmoting FITness and Health through physical activity research group (PROFITH), Department of Physical Education and Sports, Faculty of Sport Sciences, University of Granada, Grenada, Spain; 12 Department of Health Sciences, Public University of Navarra, Pamplona, Spain

**Keywords:** Type-2 diabetes, Children, Exercise, Family based lifestyle intervention program, miRNA, Ectopic fat

## Abstract

**Background:**

The global pandemic of obesity has led to an increased risk for prediabetes and type-2 diabetes (T2D). The aims of the current project are: (1) to evaluate the effect of a 22-week family based intervention program, including supervised exercise, on insulin resistance syndrome (IRS) risk in children with a high risk of developing T2D and (2) to identify the profile of microRNA in circulating exosomes and in peripheral blood mononuclear cells in children with a high risk of developing T2D and its response to a multidisciplinary intervention program including exercise.

**Methods:**

A total of 84 children, aged 8–12 years, with a high risk of T2D will be included and randomly assigned to control (*N* = 42) or intervention (*N* = 42) groups. The control group will receive a family based lifestyle education and psycho-educational program (2 days/month), while the intervention group will attend the same lifestyle education and psycho-educational program plus the exercise program (3 days/week, 90 min per session including warm-up, moderate to vigorous aerobic activities, and strength exercises). The following measurements will be evaluated at baseline prior to randomization and after the intervention: fasting insulin, glucose and hemoglobin A1c; body composition (dual-energy X-ray absorptiometry); ectopic fat (magnetic resonance imaging); microRNA expression in circulating exosomes and in peripheral blood mononuclear cells (MiSeq; Illumina); cardiorespiratory fitness (cardiopulmonary exercise testing); dietary habits and physical activity (accelerometry).

**Discussion:**

Prevention and identification of children with a high risk of developing T2D could help to improve their cardiovascular health and to reduce the comorbidities associated with obesity.

**Trial registration:**

ClinicalTrials.gov, ID: NCT03027726. Registered on 16 January 2017.

**Electronic supplementary material:**

The online version of this article (doi:10.1186/s13063-017-2117-y) contains supplementary material, which is available to authorized users.

## Background

Type-2 diabetes (T2D) is an important cause of premature death and disability as well as a costly disease affecting more than 415 million people worldwide [1]. This chronic disease is an important risk factor for developing accelerated cardiovascular disease and is the leading cause of microvascular complications such as end-stage renal disease, blindness and limb amputations. T2D was traditionally viewed as an adult-onset disease; however, over the last two decades scientific literature shows a global and dramatic increase in the incidence of T2D in youth [2], secondary to the coincident pandemic of childhood obesity [3]. The life-time risk of developing microvascular and macrovascular complications and mortality in early adulthood can be expected to be higher when the onset is already in children/adolescents rather than in adults with T2D or insulin resistance syndrome (IRS) due to the longer duration of the disease and greater duration of glycemic exposure [2, 4, 5].

As a consequence of the high rates of obesity and diabetes risk, health and scientific organizations demand urgent early preventive actions for increasing life expectancy and quality of life of overweight/obese children at a high risk of T2D [1, 6, 7]. T2D in youth is largely preventable through lifestyle intervention programs based on increasing physical activity levels, improving dietary habits and promoting a healthy body weight [8]. Likewise, overweight and obesity are the strongest risk factors for insulin resistance and T2D [9, 10]. However, long-term success rates of lifestyle intervention programs focused on T2D prevention or treatment in children are usually less than 10% [11]. The etiology of this apparent inefficiency of lifestyle intervention programs is multifactorial: lack of multidisciplinary approaches, high rates of depression or anxiety which affect the adherence to the program, insufficient involvement of the parents, etc. [12–14]. Therefore, there is a need for studies examining the effect of family based multidisciplinary intervention programs on metabolic abnormalities closely associated to T2D risk in order to give appropriate treatment/prevention options for prediabetes and T2D in overweight/obese children.

In adults, lifestyle intervention programs based on diet and physical activity level modifications have proven to be effective in decreasing the rate of the progression from glucose intolerance or prediabetes to T2D [6]. However, body mass loss achieved reducing energy intake is the cornerstone to the treatment in adults [6]. In children, energy restriction programs may compromise healthy growth and development, and increase the risk of disordered eating behaviors.

There is substantial evidence indicating that lifestyle behaviors associated to obesity and T2D are established in childhood and are difficult to modify into adulthood [15]. The adoption of healthy dietary habits is essential in the prevention of the onset of T2D in children at high risk of T2D. Consumption of sugar-sweetened beverages [16], energy-dense diets [17], reduced consumption of fiber, fruits and vegetables [18, 19], breakfast and meal skipping [20] or eating frequency [21] have been consistently associated with obesity and T2D risk. In addition, previous studies have shown the association of low physical activity levels and sedentary behaviors with insulin resistance in children [22]. Moreover, short sleep duration and poor sleep quality have been proposed as predictors of obesity and T2D onset in children and adults [23, 24].

Family based lifestyle modification interventions (i.e., targeting both the child and parent) seem to be the most efficacious treatment format for childhood obesity [25]. In addition, psychological and emotional factors are important determinants of lifestyle modifications [26] and depressive symptoms and psychological stress have negative effects on dietary behaviors and physical activity levels [27]. Moreover, overweight/obese children have an impaired psychological wellbeing compared to their non-overweight peers [28]. In this context, multicomponent lifestyle management aiming to promote healthy dietary and sleep habits, increase physical activity levels and reduce sedentary behaviors may contribute to the prevention of T2D in the short and the long term [29, 30].

Exercise is a critical component for T2D prevention and management. Likewise, regular physical activity improves insulin sensitivity in diabetic or prediabetic children [31]. Previous studies have shown that exercise training reduces insulin resistance in overweight/obese youth, even without body mass loss [32]. Interestingly, Davis et al. reported that the benefit of aerobic training on insulin resistance was dose-dependent in overweight children regardless of sex and race [33]. However, although exercise is recommended for overweight/obese children, optimal exercise prescription describing type (aerobic training, resistance training or combined training) and intensity (moderate or vigorous) of exercise needed to reduce T2D risk in children is unclear.

Obesity, insulin resistance and T2D are caused by a combination of genetic, epigenetic and environmental factors such as diet and exercise. During the last few years, a growing number of studies demonstrate that small, non-coding, ribonucleic acid (RNA) molecules that function as regulators of gene expression, microRNAs (miRNAs), may be involved in the pathogenesis of obesity, diabetes and related metabolic disorders [34, 35]. Several studies observed that miRNAs play a significant role in insulin production and secretion, pancreatic islet development and β-cell differentiation, and that they are implicated in several aspects of glucose homeostasis and lipid metabolism associated with the pathogenesis and progress of T2D and obesity [36–38]. The stability and presence of miRNAs in a variety of readily accessible cell types including body fluids, as well as their often tissue- and disease-specific expression, and the possibility to measure them with high sensitivity and specificity, render them a potential source of clinical biomarkers of T2D risk [39]. Moreover, miRNAs reflect individual biological adaptations to physiological changes or environmental exposures such as diet or exercise. Likewise, measurement of miRNAs presents an opportunity to evaluate biological changes associated with lifestyle interventions for the reduction of metabolic disorders associated with T2D risk such as insulin resistance, ectopic fat accumulation, inflammation, etc. The majority of the studies aiming to identify miRNAs as biomarkers of obesity and T2D have been conducted in small samples of adults. Previous reports observed changes in circulating miRNA profile after metformin therapy [38] or exercise in prediabetic adults [40]. In obese children, several miRNAs have been proposed as biomarkers of endothelial dysfunction and dyslipidemia [41]. As far as we are aware, there are no previous studies identifying the profile of miRNAs in children at a high risk of T2D and its response to a family based multidisciplinary intervention program including exercise.

Therefore, the primary aims of the PREDIKID (Prevention of Diabetes in Kids) study are: (1) to evaluate the effect of a 22-week, family based, multidisciplinary intervention program including exercise on IRS in children with a high risk of developing T2D and (2) to identify the profile of microRNA in circulating exosomes and in peripheral blood mononuclear cells in children with a high risk of developing T2D and its response to a family based multidisciplinary intervention program including exercise.

## Methods

### Design

**Fig. 1:**
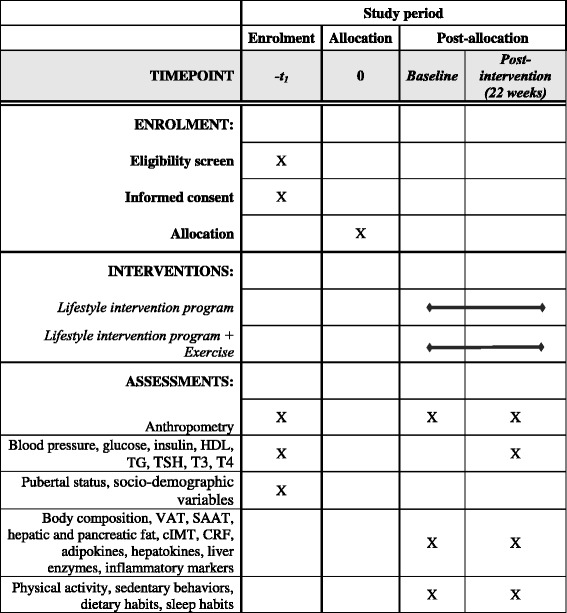
Standard Protocol Items: Recommendations for Interventional Trials SPIRIT Figure. *cIMT* carotid intima media thickness; *CRF* cardiorespiratory fitness; *HDL* high-density lipoprotein; *SAAT* subcutaneous abdominal fat; *TG* triglycerides; *VAT* visceral abdominal fat

The PREvention of DIabetes in KIDs (PREDIKID) study is a randomized clinical trial (RCT) (ClinicalTrials.gov, ID: NCT03027726) (see Additional file 1 and Fig. 1 for the associated Standard Protocol Items: Recommendations for Interventional Trials (SPIRIT) Checklist and Figure, respectively). Children will be randomly allocated to the control group or the exercise group and will be followed for 22 weeks. All the families included in the study will participate in a lifestyle education and psycho-educational program conducted by a multidisciplinary team of health care professionals (physicians, nutritionists and dieticians, psychologists and exercise specialist) and scientists. As both groups will receive the same lifestyle education and psycho-education program, differences in changes in outcome variables will be due to the additional effect of the exercise intervention. This study was reviewed and approved by the Ethics Committee of Clinical Investigation of Euskadi. All parents or legal guardians will sign an informed written consent and all the children will give their assent before being enrolled in the study. The informed consent will be obtained by the pediatricians participating in the recruitment.

#### Participants and selection criteria

Participants will be recruited by pediatricians from the Pediatric Endocrinology Unit of the University Hospital of Txagorritxu and from primary care clinics (Vitoria-Gasteiz, Spain). Children aged between 8 and 12 years, meeting the international criteria for classification of T2D risk [1, 42], and having at least one parent or caregiver willing to participate in the program sessions, will be included (Table 1).

**Table 1:** Eligibility criteria for the PREvention of DIabetes in KIDs (PREDIKID) study

Inclusion criteria
Aged 8–12 years
Criteria for the classification of risk of T2D [1]^a^
1. Obesity status according to the World Obesity Federation [24] 2. Overweight and the presence of at least one of the following metabolic abnormalities: 2.1. Family history of T2D in first- or second-degree relative; Signs of insulin resistance or conditions associated with insulin resistance (acanthosis nigricans, HOMA > 2.5, glucose between 100 and 125 mg/dL; Hypertension (systolic blood pressure ≥ 130 mmHg and/or diastolic blood pressure ≥ 85 mmHg); Dyslipidemia (HDL < 40 mg/dL, TG > 150 mg/dL); 2.2. Ethnic minority with higher risk
To have at least one parent or caregiver willing to participate in the program sessions
Exclusion criteria
Not available for assessment and intervention sessions
Medical conditions or medications that would affect study results or limit physical exercise

*PREDIKID* PREvention of DIabetes in Kids. ^a^According to the International Diabetes Federation [1]. *T2D* type-2 diabetes; *TG* triglycerides; *HDL* high-density lipoprotein; *HOMA* homeostatic model assessment

A pre-screening appointment will be scheduled with the investigation team to assess eligibility, family needs and commitment. Parents will provide the child’s and family health history and detailed contact information. After completion of the informed consent process, children will undergo anthropometric screening (height, body mass and waist circumference), complete physical examination and blood sampling, and will be selected for inclusion in the study if they meet the inclusion criteria (Table 1). Every child meeting the inclusion criteria will go through maximal exercise testing guarantee. Children with any medical condition that could affect the results of the study or that limits physical activity will be excluded.

#### Sample size calculation

Insulin resistance is the primary outcome of this study. We expect that pre-post intervention differences in our design consisting in two experimental groups, control and exercise group, will have a size effect (Cohen’s *d*) of 0.7 for insulin resistance (i.e., homeostatic model assessment, HOMA) (*N* = 34 in each group, 80% power and *α* of 0.05). These effect size estimates are based on previous studies performed in overweight children with similar age range and a with intervention programs including supervised exercise [33, 43, 44]. We observed in one previous study, in which the effect of the same intervention program was tested on hepatic fat in overweight/obese children, losses to follow-up of approximately 7% (data not shown). Previous studies have reported losses to follow-up of between 4 and 17%. Assuming a maximum loss of follow-up of 20%, we decided to recruit a total of 84 children (50% girls), 42 children for each group.

#### Randomization

**Fig. 2:**
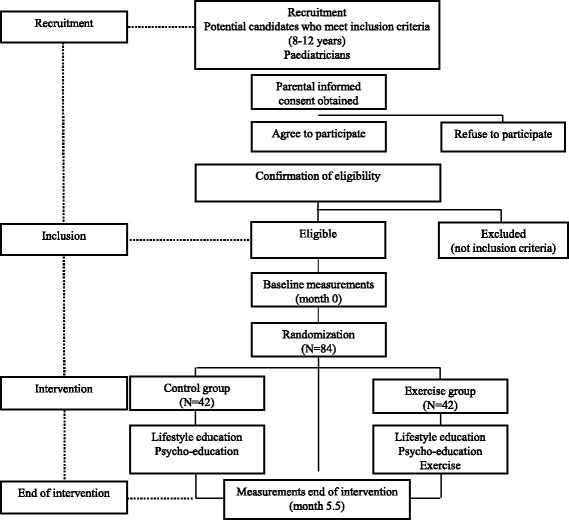
Planned study design

Eligible participants will be randomly assigned after completing the baseline measurements to either the control group or the or exercise group (Fig. 2). Randomization of the participants into the control or exercise group will be done using Statistical Package for Social Sciences (IBM SPSS Statistics for Windows, version 21, Armonk, NY, USA) by an independent researcher. Assessment staff will be blinded to participant randomization assignment. Participants and their parents or guardians will be explicitly informed of the group to which they will be assigned, as well as of the study hypotheses. As exercise is potentially beneficial for the health of the children recruited in this trial, the waiting list control group strategy will be used, which means that the control group will receive the same exercise program when the post-intervention evaluations are finished.

#### Family based healthy lifestyle education and psycho-educational intervention

Families in the control and the exercise groups will attend both the healthy lifestyle education and psycho-education program once every 2 weeks (11 sessions over 22 weeks) for 90 min (45 min in the healthy lifestyle education program and 45 min in the psycho-educational intervention). The design of the programs has been published elsewhere [45]. Briefly, the sessions of the healthy lifestyle education and psycho-educational interventions will be developed simultaneously and delivered to both parents (or caregivers) and children, separately.

The family based healthy lifestyle education program will be conducted by experienced nutritionists. The aim of the program will be in promoting changes in lifestyle behaviors strongly associated to obesity and T2D risk: promoting healthy dietary habits (reducing the intake of sugar, sugar-sweetened beverages and energy-dense foods, enhancing the daily consumption of fruits and vegetables, promoting breakfast and eating five meals/day), increasing regular physical activity levels, reducing time spent in sedentary behaviors and promoting sleep hygiene and adequate sleep duration.

The aims of the psycho-educational program will be to provide parental skills to optimize the family environment in order to make positive changes in their lifestyles and to learn assertive communication skills. The program will also provide skills to children for managing the emotions and feelings that they are experiencing and to improve their self-esteem and psychological wellbeing.

#### Exercise training intervention

The exercise group will do exercise 3 days/week, 90 min per session, over a 22-week period. The program will be open to the families 5 days per week to choose a total of 3 days/week. Sessions will be designed and supervised by exercise specialists. The design of the exercise program has been published elsewhere [45]. Briefly, the program consists of cardiovascular endurance (60 min) and muscle strength exercises (10 min). The sessions also include 10 min of warm-up period, 5 min for giving instructions and 5 min of cool-down period at the end, consisting mainly of stretching exercises. Moderate to vigorous intensity aerobic workout is the main part of the exercise session (60 min) and consists of games, circuits and start-and-stop activities. The emphasis of the program is on intensity doing special emphasis on high intensity activities (above 75% of maximal heart rate (HRmax) obtained from either the cardiopulmonary exercise test in the laboratory or the 20-m shuttle run test) and enjoyment. Participants will wear a heart rate monitor (Polar RS300X) during the exercise sessions to ensure achievement of the target heart rate zone. Heart rate monitors will be programmed according to individual percentage of HRmax as follows: (1) very light intensity: from 50 to 57% of HRmax, (2) light intensity: from >57 to 64% of HRmax, (3) moderate intensity, from >64 to 76% HRmax, (4) high intensity or vigorous: from >76 to 96% HRmax and (5) maximal intensity: > 96% of HRmax.

### Participant retention and adherence

The success of the intervention is strongly dependent on enjoyment and active participation. In order to motivate children’s active participation, the staff will use several strategies to celebrate success achieving the proposed objectives in both the healthy lifestyle education and the exercise programs: celebrate and recognize their efforts, reward with smiley emoticons, etc. Children who complete the program successfully will be rewarded with a certificate of completion. The attendance to the lifestyle education and psycho-education programs and of the exercise program will be recorded. Children will be marked as absent when: (1) the child does not attend the exercise session or does not participate in the proposed activities and (2) the child and at least one parent/caregiver do not attend one session of the healthy lifestyle education and psycho-educational programs.

### Outcome measures for the first objective

IRS and the risk of T2D is characterized by excess adiposity, low levels of insulin sensitivity, dyslipidemia and hypertension. In the current project, the primary outcome is insulin resistance. Secondary outcome variables include total, abdominal and visceral adiposity, ectopic fat (hepatic and pancreatic fat), cardiorespiratory fitness, cardiovascular disease risk factors and inflammation markers, hepatokines and adipokines (Table 2). Primary and secondary outcomes will be evaluated at baseline and repeated after 22 weeks of intervention (Fig. 2). Post-intervention measurements will be scheduled within 3 days following the last healthy lifestyle program session or exercise session in the control or in the exercise group, respectively.

**Table 2:** Summary of the methodology of primary and secondary outcome measurements and potential confounders in the PREvention of DIabetes in KIDs (PREDIKID) study

Measure	Methodology
Primary outcomes	
Insulin resistance	Homeostatic model assessment (HOMA), biochemical analyses
Expression of miRNA in circulating exosomes and white blood cells	RNA-seq methodology
Secondary outcomes	
Physical measures	
Body mass (kg)	Scale
Height (cm)	Stadiometer
Waist circumference (cm)	Non-elastic tape
Blood pressure (mmHg)	Oscillometric monitor device
Body fat (%)	Dual X-ray absorptiometry
Abdominal adiposity (g)	Dual X-ray absorptiometry
Visceral abdominal adiposity (cm^2^)	Magnetic resonance imaging
Hepatic fat (%)	Magnetic resonance imaging
Pancreatic fat (%)	Magnetic resonance imaging
Carotid intima medial thickness (mm)	Doppler ultrasonography
Biochemical measures	
Glucose (mg/dL)	Enzymatic spectrophotometry
Insulin, leptin, adiponectine, fetuin-A, FGF21	Enzyme-Linked Immunosorbent Assay
Hemoglobin A1c	Enzymatic tests
Total cholesterol, HDL- and LDL-cholesterol (mg/dL)	Enzymatic spectrophotometry
Triglycerides (mg/dL)	Enzymatic spectrophotometry
Alanine aminotransferase (U/L)	Enzymatic tests
Aspartate aminotransferase (U/L)	Enzymatic tests
Gamma glutamyl transferase (U/L)	Enzymatic tests
C-reactive protein (g/dL)	Enzyme immunoassay
TSH, T3, T4	Radioimmunoassay
Uric acid (mg/dL)	Enzymatic spectrophotometry
Urea, bilirubin	Enzymatic spectrophotometry
Cardiorespiratory fitness	20-m shuttle run test Cardiopulmonary exercise test
Dietary assessment	24-h recalls and food frequency questionnaires
Physical activity assessment	Accelerometry and questionnaires
Sleeping habits	Accelerometry and questionnaires
Sedentary behaviors	Questionnaires
Potential confounders	
Pubertal development (Tanner stage)	Physical examination
Sociodemographic variables	
Socioeconomic status	Questionnaires
Neonatal variables	Health booklets and questionnaires
Family medical history	Questionnaires
Demographic characteristics	Questionnaires

*PREDIKID* Prevention of Diabetes in Kids; *FGF21* fibroblast growth factor 21; *HDL* high-density lipoprotein; *LDL* low-density lipoprotein; *miRNA* micro RNA; *TSH* thyroid stimulating hormone; *T3* triiodothyronine; *T4* free thyroxine

#### Primary outcome measure: insulin resistance

HOMA has been shown to be the more reliable method to assess insulin resistance in children [46]. The computer-generated HOMA will be used [47].

#### Secondary outcomes

##### Anthropometry and blood pressure

Body mass (SECA 760), height (SECA 220) and waist circumference (SECA 200) will be measured following standard protocols at least twice until consistent measures are obtained and, thereafter, Body Mass Index (BMI) and waist to height ratio will be calculated. Systolic and diastolic blood pressure measurements will be performed following the recommendations for children [48] using an arm blood pressure oscillometric monitor device (OMRON® M6).

##### Total, abdominal and visceral adiposity

In children, the role of total, abdominal and visceral fat accumulation in the development of IRS, and ultimately T2D, is well established. One recent report observed that every additional 1% of adiposity at 8–10 years decreased insulin sensitivity by 2.9% 2 years later [22]. Total and abdominal adiposity will be measured by dual energy X-ray absorptiometry (HOLOGIC, QDR 4500 W). Visceral adiposity will be measured by magnetic resonance imaging using a 1.5-T system (MAGNETOM Avanto, Siemens Healthcare, Erlangen, Germany) equipped with a phased-array surface coil and a spine array coil. Automated visceral fat measurement will be achieved by image processing (Matlab, Matworks Inc., Natick, MA, USA).

##### Ectopic fat

Excess adiposity is associated with an accumulation of fat in multiple organs and tissues. Fat accumulation in the liver and pancreas promotes a pro-inflammatory state that may increase the risk of insulin resistance and T2D. Likewise, ectopic fat accumulation seems to be an important predictor of the onset of T2D in adolescents [49]. Insulin sensitivity was 55% lower in adolescents with hepatic steatosis compared to those without fatty liver disease. Few studies have examined fat accumulation in the pancreas and its relationship with insulin resistance and T2D. Pancreatic steatosis seems to be associated with prediabetes in children increasing the risk of T2D and cardiovascular disease [50–52]. Nevertheless, other studies reported no significant associations of pancreatic fat and β-cell dysfunction [53]. There are no previous studies examining the effect of either a lifestyle modification program or an exercise program on pancreatic fat. Hepatic and pancreatic fat will be measured by magnetic resonance imaging using a 1.5-T system (MAGNETOM Avanto, Siemens Healthcare, Erlangen, Germany) equipped with a phased-array surface coil and a spine array coil.

##### Cardiorespiratory fitness

Cardiorespiratory fitness is an independent predictor of T2D in adults [54]. In youths, there is evidence indicating a negative association between cardiorespiratory fitness and insulin resistance [55]. Moreover, previous reports suggested that that the relationship between cardiorespiratory fitness and T2D may be mediated by an increased susceptibility to fat deposition in liver and pancreas, increasing thereby insulin resistance [49, 56]. Cardiorespiratory fitness will be assessed by two different tests as published elsewhere [45]: (1) the 20-m shuttle run test [57]. We will use the equation reported by Léger et al. [57] to estimate the maximum oxygen consumption (VO_2max_, ml/kg/min) from the 20-m shuttle run test scores and (2) direct cardiopulmonary exercise progressive incremental treadmill test using the modified American College of Sports Medicine protocol with respiratory gas analysis to exhaustion [58].

##### Carotid intima-media thickness

Early changes of atherosclerosis can be detected assessing carotid intima-media thickness (cIMT). In adolescents with T2D, Shah et al. reported that every 1% increase in hemoglobin A1c (HbA1c) or each year increase of the duration of the disease was associated with 30% higher cIMT [59]. In children with obesity, cIMT was higher in those with prediabetes than in their counterparts with normal glucose tolerance [60]. cIMT will be measured by ultrasound (General Electric, Logic S8 model) according to international recommendations. All studies will be done following a standardized scanning protocol for the right and left common carotid arteries. The common carotid artery bulb will be identified and the segments of common carotid arteries 1–2 cm proximal to the bulb region will be scanned. During the end of the diastole cIMT will be measured in both carotids four times and mean cIMT and maximal cIMT will be calculated. Also in telediastole, two sides of maximal diameter of the common carotid, bulb and internal carotid will be measured.

##### Inflammation and biochemical cardiovascular disease risk factors

The obesity-associated chronic inflammatory process participates in the onset of T2D, although the underlying mechanisms are not well understood [61, 62]. Several studies have suggested that inflammatory cytokines may influence insulin sensitivity, both directly and also indirectly mediated, by adipokines, such as leptin and adiponectin, and hepatokines such as fetuin-A and fibroblast-21 growth factor (FGF-21) [62–65]. Biochemical variables will be measured from venous fasting blood samples obtained from each child by experienced nurses. Blood samples will be immediately centrifuged, aliquoted and stored at −80 °C or below. This permits the measurement of a full set of cardiovascular markers including lipid profile (total cholesterol, HDL- and LDL-cholesterol and triglycerides), glucose, insulin, HbA1c, cytokines (e.g., tumor necrosis factor alpha and interleukin (IL)-6) adipokines (e.g., leptin and adiponectin), hepatokines (fetuin-A and FGF-21), liver enzymes (alanine aminotransferase, aspartate aminotransferase and gamma-glutamyl transferase), C-reactive protein and uric acid. ELISA kits, Western blots and High Performance Liquid Chromatography-Mass Spectroscopy will be used to perform these analyses.

##### Physical activity, sedentary behavior, dietary assessment and sleep

We will assess all these lifestyle behaviors both at the beginning and the end of the intervention. Children will wear an accelerometer on the non-dominant wrist (ActiSleep, Actigraph, Pensacola, FL, USA) for seven consecutive days for 24 h to record physical activity intensity levels and patterns, as well as sleeping habits and participants will also complete a diary log. Sedentary behaviors, such as watching TV, playing computer games, playing video games or phone games and surfing the Internet, will be self-reported by the children using validated questionnaires [66]. Dietary intake will be evaluated by two non-consecutive 24-h recalls within a period of 7 days by nutritionists and a self-reported semi-quantitative food frequency questionnaire.

### Outcome measures for the second objective

Recently, miRNAs have emerged as key regulators of gene expression. T2D is associated with changes in the level of several miRNAs in pancreatic β-cells as well as in tissues such as liver, skeletal muscle or adipose tissue [67, 68]. Moreover, miRNA expression seems to participate in the regulation of the IRS mediated by the regulation of inflammatory processes [39]. As has been mentioned in previous sections, the second primary aim of the present work is to characterize the miRNA species present in circulating white blood cells and exosomes, before and after the intervention program is applied. Our initial hypothesis is that the application of this program will have a direct impact on the miRNA species present in blood cells and exosomes, and that these changes will influence the physiological effects elicited by the exercise program.

#### Extraction and isolation of miRNAs from white blood cells and plasma

Exosomes will be obtained from 1 ml of plasma using the Total Exosome Isolation Kit (Thermo Fisher Scientific) following manufacturer’s protocols. RNA molecules will be isolated from white blood cells (buffy coat) and exosomal fractions using Qiagen miRNeasy kit (Qiagen, Hilden, Germany) according to the manufacturer’s instructions. Once isolated, RNA will be quantified using a fluorimetric method.

#### Analysis and validation of miRNAs

Species of miRNA present on samples of individual subjects, before and after the intervention program, will be analyzed using RNA-seq methodology on Illumina’s MiSeq Next Generation Sequencing system. This workflow allows one to fully characterize the miRNA profile present in the samples analyzed (exosomes and white blood cells) and to quantify the expression levels of different miRNA species. In this sense, effects of the intervention program on the miRNA profile and on expression level of specific species can be inferred. Briefly, Illumina’s TruSeq Small RNA Sample preparation kit will be used to generate sequencing libraries, from 1 μg of total RNA isolated from previous samples (either exosomes or white blood cells). Only 145 to 160 bp libraries will be selected for sequencing, as they include miRNA species (22–30 bp plus adapters). Sequencing reactions will be performed on Illumina’s MiSeq Reagent Kit V3, which supports to multiplex up to 10 samples on a single run, and single reads of 50 nt in length. Analysis of results, FASTQ files generation and sequence alignment will be performed using specific software packages (MiSeq Reporter, Bowtie, SAMtools, DESeq2 and miRDeep).

### Confounding variables

#### Puberty stage

At baseline, the pediatrician will evaluate Tanner staging by direct-examination breast palpation in girls and testicular measurement by orchidometer in boys.

#### Sociodemographic variables

Information about socioeconomic status, demographic characteristics and family medical history of obesity, diabetes, dyslipidemia and hypertension will be collected. Socioeconomic status will be evaluated using The Family Affluence Scale [69] and parental education level and parental occupation. As demographic characteristics, date of birth, sex, ethnicity and family structure will be recorded.

### Assessment of side effects

All adverse effects or health problems attributable to the testing or exercise sessions will be recorded.

### Data management and monitoring

Participants’ data will be identified only by a study number (starting with 001) and only the principal investigator and study coordinator will have access to identifiers that can link the data to the individual participant. Participants who drop out will be noted and their reasons documented. Data will be entered and stored on a standalone computer into excel and SPSS data files, and the information will be password protected. We will assure the quality of data entry by random checking of the data entered. Study data is only accessible by the researchers involved in the trial, and only the principal investigator and investigators in the project will have access to the data for analysis. The investigators will monitor that the informed consent process is conducted appropriately and that informed consent was obtained prior to proceeding with any study procedures. Only participants who meet study eligibility criteria will be enrolled. Data will be kept for 10 years after the research is completed and all data (electronic and hard copy) will be destroyed after the storage period. There is no data monitoring committee due to the characteristics of the current study.

### Data analyses plan

All outcome variables will be checked for normality and results will be expressed as mean and standard deviation (SD) or median and ranges. Multiple regression analyses will be performed to examine the potential independent predictors of T2D risk. At baseline, differences in continuous variables between groups (control group vs. exercise group) will be analyzed by Student’s *t* test or the Mann-Whitney *U* test, as appropriate. Differences in categorical variables at baseline between the two groups will be explored by chi-squared tests. Repeated-measured analysis of variance will be used to assess the effect of exercise (time (pre-post 22-weeks’ intervention) × group interactions) on the primary (insulin resistance) and secondary study (adiposity, ectopic fat, cardiorespiratory fitness, cardiovascular disease risk factors, inflammation markers, etc.) outcomes and the intention-to-treat principle will be applied. For sensitivity analyses, we will analyze the effect of the adherence to the program on the results of the intervention.

### Data dissemination plan

The principal investigator will inform the parents about their child’s health before and after the intervention program. The main results of the trial will be communicated to the health care professionals (pediatricians) in Vitoria-Gasteiz and other relevant groups (e.g., university academics), as well as to the public. Scientific articles will be also written.

## Discussion

The worldwide obesity pandemic and the increasing prevalence of T2D at younger ages are important public health issues with both individual and social costs. Likewise, research focusing on the prevention of T2D in pre-adolescent children is a high priority for public health. Scientific associations emphasize the need for evidence-based adiposity and IRS control treatments that may reduce T2D risk in children at a high risk [6].

The primary aim of the PREDIKID project is to test the effect of a multidisciplinary program including supervised exercise in the reduction of IRS in pre-adolescents at a high risk to develop T2D that could serve as preventive model of T2D in childhood. The families participating in the study will receive an educational program including lifestyle, psychological and emotional aspects that the scientific literature implicates in the development and progression of obesity and T2D risk in childhood. Moreover, the exercise intervention of the PREDIKID study will obtain objective data about the effect of high intensity exercise on metabolic variables closely associated to T2D risk in children, such as insulin resistance, HbA1c, ectopic fat, total, abdominal and visceral adiposity, cIMT, cardiorespiratory fitness, adipokines, hepatokines and cytokines, blood pressure or lipid profile. We will examine clinical outcomes intimately associated with T2D using innovative technology and reference methods. Likewise, the PREDIKID project will help to better understand the relationship between hepatic and pancreatic fat and IRS and risk of T2D in children. In addition, the current study may offer relevant clinical information about the efficacy of a family based multidisciplinary intervention program that includes exercise on reducing ectopic fat, IRS and cardiovascular disease risk factors in children at a high risk of T2D.

The pathophysiology of childhood obesity and associated comorbidities, such as T2D, is not well established. To date it is not clear why some children develop IRS and T2D, while others show the metabolically healthy phenotype [70]. Biomarkers for early detection of children at risk of T2D would greatly improve the care of children with overweight/obesity. Hence, there is a need to search for clinically relevant markers with prognostic value for intervention success. Recently, miRNAs have been proposed as promising biomarkers in terms of both diagnosis and prognosis of obesity-related comorbidities [71, 72]. The PREDIKID project will help to identify miRNAs potentially associated with early onset of IRS in children with overweight/obesity. Moreover, the differential expression and responses in circulating miRNAs between the control group and the exercise group may help to understand the underlying mechanisms of the effect of exercise on IRS, as well as to predict individual variability to the intervention in overweight/obese children.

### Trial status

The recruitment started on March 2017, and is expected to finish in September 2018.

## Additional file

Additional file 1: Recommendations for Interventional Trials (SPIRIT) Checklist. (DOC 122 kb)

## Abbreviations

BMI: Body Mass Index
cIMT: Carotid intima-media thickness
HDL: High-density lipoprotein
HOMA: Homeostatic model assessment
HRmax: Maximal heart rate
IRS: Insulin resistance syndrome
LDL: Low-density lipoprotein
miRNAs: MicroRNAs
PREDIKID: Prevention of Diabetes in Kids
T2D: Type-2 diabetes
